# Loss of USP18 in microglia induces white matter pathology

**DOI:** 10.1186/s40478-019-0757-8

**Published:** 2019-07-04

**Authors:** Marius Schwabenland, Omar Mossad, Adam G. Peres, Franziska Kessler, Feres Jose Mocayar Maron, Laura-Adela Harsan, Thomas Bienert, Dominik von Elverfeldt, Klaus-Peter Knobeloch, Ori Staszewski, Frank L. Heppner, Marije E. C. Meuwissen, Grazia M. S. Mancini, Marco Prinz, Thomas Blank

**Affiliations:** 1 Institute of Neuropathology, Faculty of Medicine, University of Freiburg, Breisacher Str. 64, 79106 Freiburg, Germany; 2 Faculty of Biology, University of Freiburg, Freiburg, Germany; 3 Department of Radiology, Medical Physics, University Medical Center Freiburg, Faculty of Medicine, University of Freiburg, Freiburg, Germany; 4 Engineering Science, Computer Science and Imaging Laboratory (ICube), Integrative Multimodal Imaging in Healthcare, CNRS, University of Strasbourg, Strasbourg, France; 5 Department of Neuropathology, Charité - Universitätsmedizin Berlin, corporate member of Freie Universität Berlin, Humboldt-Universität zu Berlin, and Berlin Institute of Health, Charitéplatz 1 (Virchowweg 15), 10117 Berlin, Germany; 6 Department of Clinical Genetics, Erasmus University Medical Center, 3015 GD Rotterdam, the Netherlands; 7 Signalling Research Centres BIOSS and CIBSS, University of Freiburg, Freiburg, Germany; 8 Center for NeuroModulation, Faculty of Medicine, University of Freiburg, Freiburg, Germany; 9 Cluster of Excellence, NeuroCure, Charitéplatz 1, 10117 Berlin, Germany; 10 German Center for Neurodegenerative Diseases (DZNE) Berlin, 10117 Berlin, Germany

**Keywords:** Microglia, Type I interferon, Usp18, White matter, Phagocytosis, Corpus callosum, Behavior, Magnet resonance spectroscopy, Microgliosis

## Main text

Ubiquitin specific protease 18 (USP18) is a major negative regulator of the type 1 interferon (IFN) pathway. In a recent publication we showed that USP18 is a key molecule imposing microglial quiescence specifically in the white matter [7]. USP18 is a negative regulator of the type 1 interferon (IFN) pathway [9]. Microglia lacking *Usp18* exhibited constitutive activation of type I IFN signaling pathways resulting in markedly elevated expression of multiple interferon-stimulated genes (ISGs) [7]. Additionally, *Usp18*-deficient brains exhibited clusters of microglia in the white matter that strongly resembled the neuropathological state in several human microgliopathies. Human diseases in which microgliopathies play a primary role comprise Nasu-Hakola disease [14], hereditary diffuse leukoencephalopathy with spheroids (HDLS) [15] and Pseudo-TORCH syndrome (PTS), including Aicardi–Goutières syndrome [12]. One might speculate that activated microglia in the white matter induce white matter abnormalities with functional consequences. However, there were no cells which had taken up myelin in young adult mice as seen by luxol fast blue–PAS (LFB–PAS) histology (unpublished data). Myelin uptake by other cells, like macrophages, would have been indicative of myelin damage. That is why we now characterized conditional myeloid-specific *Usp18* deficient mice in more detail.

**Fig. 1:**
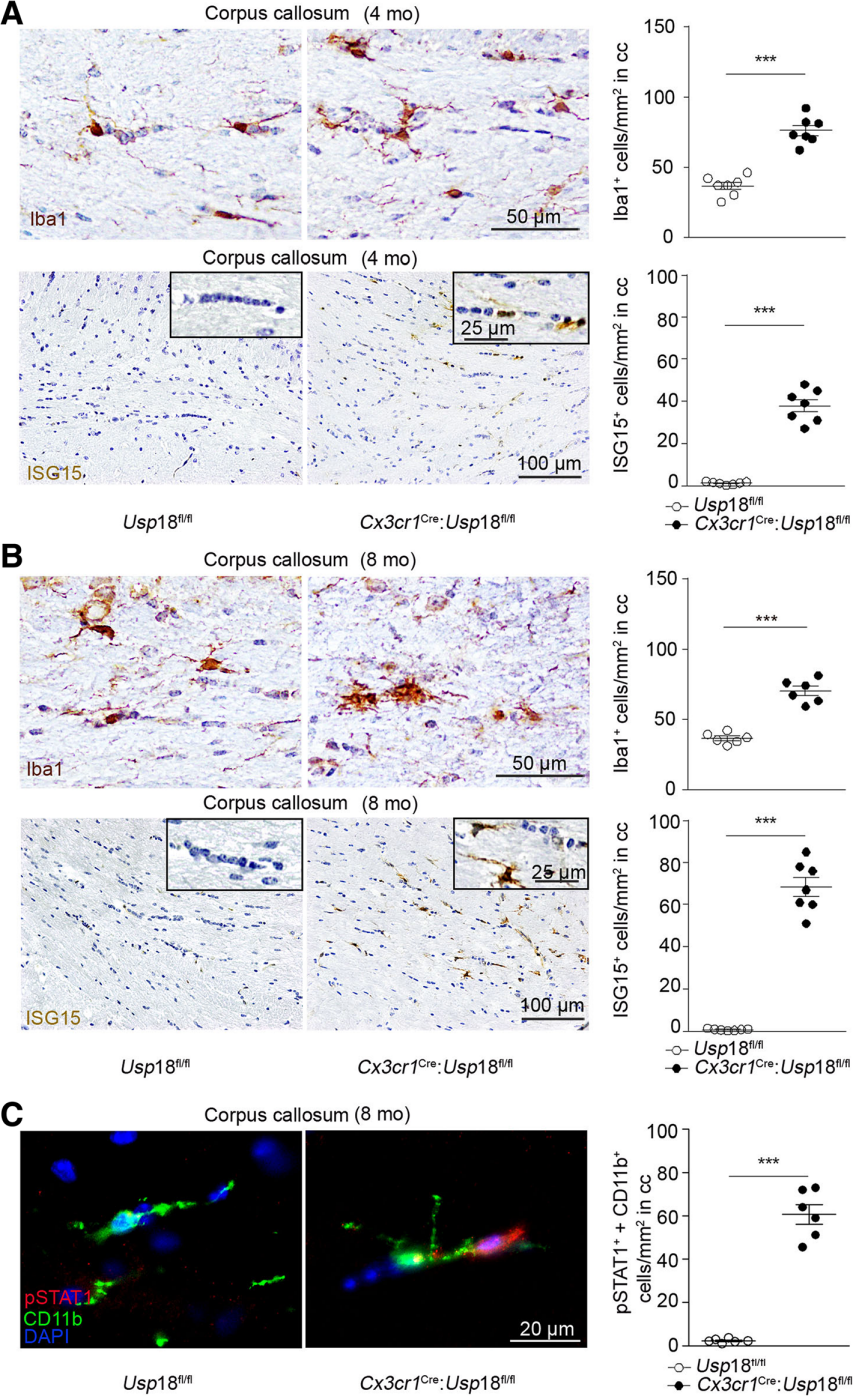
Microgliosis in corpus callosum of *Cx3cr1*^*Cre*^:*Usp18*^*fl/fl*^ mice. **a**, **b** Histology of corpus callosum in the cerebrum of adult *Usp18*^*fl/fl*^ and *Cx3cr1*^*Cre*^:*Usp18*^*fl/fl*^ mice at 4 (**a**) and 8 months of age (**b**). Primary antibodies against Iba1 and ISG15 were used. To quantify the number of Iba1^+^ or ISG15^+^ cells at least six mice per genotype and 5 sections per mouse from two independent experiments were counted. Quantification of cells is shown next to the respective histological images. Significant differences were determined by an unpaired *t*-test or Mann-Whitney *U*-test and marked with asterisks (***P < 0.001 versus control littermates). Bars represent means ± S.E.M. Scale bars = 25 μm, 50 μm, 100 μm. **c** Immunohistochemistry for phosphorylated STAT1 (pSTAT1, red), CD11b (green) and DAPI (blue) in the corpus callosum of 8- month old *Usp18*^*fl/fl*^ and *Cx3cr1*^*Cre*^:*Usp18*^*fl/fl*^ mice. Scale bar: 20 μm. Quantification of pSTAT1^+^CD11b^+^ cells is shown next to the respective histological images. Each symbol represents one mouse. Error bars represent S.E.M. Significant differences are determined by an unpaired *t*-test and marked with asterisks (***P < 0.001)

**Fig. 2:**
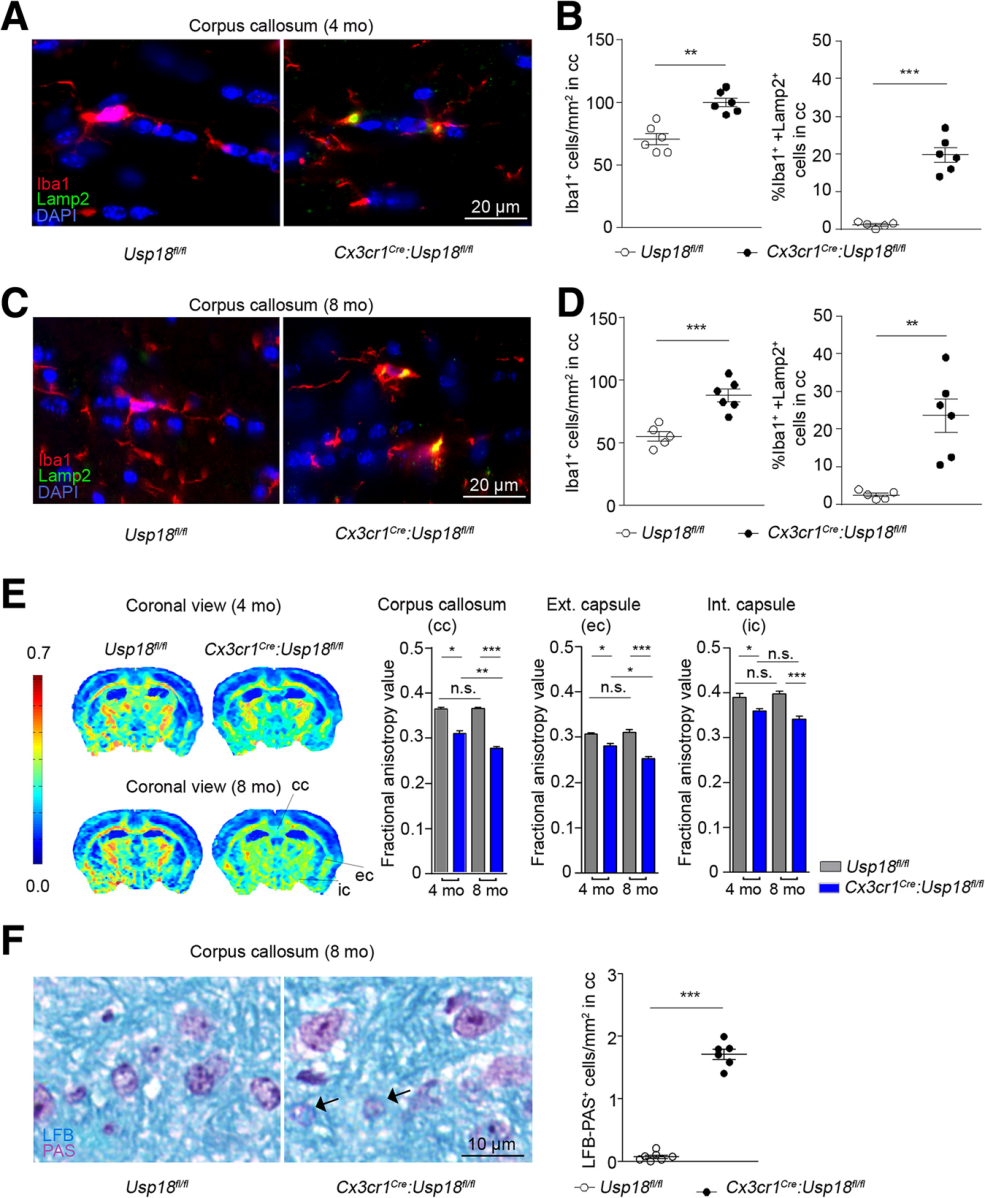
USP18-deficient microglia reduces structural integrity in corpus callosum. **a** Immunofluorescent histochemistry for Iba1 (red), Lamp2 (green) and DAPI (blue) in the corpus callosum of 4 months and 8 months (**c**) old *Usp18*^*fl/fl*^ and *Cx3cr1*^*Cre*^:*Usp18*^*fl/fl*^ mice. Scale bar: 20 μm. Quantification of Iba1^+^ and percentage of Iba1^+^Lamp2^+^ cells is shown next to the respective histological images (**b**, **d**). Each symbol represents on mouse. Error bars represent s.e.m. Significant differences are determined by an unpaired *t*-test and marked with asterisks (**P < 0.01, ***P < 0.001). **e** DTI was performed on 4 and 8 months old *Usp18*^*fl/fl*^ and *Cx3cr1*^*Cre*^:*Usp18*^*fl/fl*^ mice to measure the FA of the corpus callosum. Tensor images were collectively acquired in several horizontal planes from + 2.0 to − 4.0 mm from the bregma, with an interplane distance of 0.5 mm (*Usp18*^*fl/fl*^, *n* = 6; *Cx3cr1*^*Cre*^:*Usp18*^*fl/fl*^, *n* = 4). Heat maps of the FA values showing the average (of all *Usp18*^*fl/fl*^ and *Cx3cr1*^*Cre*^:*Usp18*^*fl/fl*^ animals) of one plane from each group (from anterior to posterior). Warm colors indicate fiber tracts with strong diffusion coherence. For both age groups the FA values were significantly reduced in *Cx3cr1*^*Cre*^:*Usp18*^*fl/fl*^ mice in comparison to *Usp18*^*fl/fl*^ mice. Approximate locations of the regions of interest (ROIs) are indicated. Data are means ± SEM. (*P < 0.05, **P < 0.01, ***P < 0.001, n.s. = non-significant). Statistical significance was determined using multiple *t* tests corrected for multiple comparisons using the Holm-Sidak method with a = 0.05. **f** Histological analysis by luxol fast blue–PAS (LFB–PAS) in 8-month-old *Usp18*^*fl/fl*^mice and *Cx3cr1*^*Cre*^*:Usp18*^*fl/fl*^ littermates. Representative of *n* = 6 *Usp18*^*fl/fl*^ and *n* = 7 *Cx3cr1*^*Cre*^*:Usp18*^*fl/fl*^ mice. Circles represent individual mice. Unpaired two-tailed *t*-test

We know that *Usp18* transcripts are highly expressed in unstimulated white matter microglia with only negligible expression levels in other CNS cells [7]. In a previous study, we have confirmed by PCR analysis that *Cx3cr1*^*Cre*^:*Usp18*^*fl/fl*^ mice have an *Usp18* deletion in microglia but not in neuroectodermal cells of the CNS. These mice displayed a significant increase of Iba1^+^ microglia cell numbers in several white matter regions including the corpus callosum as young adult mice [7]. This microgliosis persisted with increasing age and was detectable even in 4- and 8-month old mice (Fig. 1a, b). *Usp18*-deficient microglia exhibit constitutive expression of IFN target genes and fail to downregulate IFN-induced genes because the termination of type I IFN signaling is severely impaired. This became evident by the increase in ISG15 positive cells in the corpus callosum (Fig. 1a, b) and the elevated phosphorylation of STAT1 in *Usp18*-deficient microglia when compared to *Usp18*^*fl/fl*^ mice (Fig. 1c). We next investigated animals at later ages than before by immunostainings against lysosome-associated membrane protein-2 (LAMP2) as a marker of phagocytosis [4]. We found increased LAMP2 positive signals in microglia, which were localized in the corpus callosum of *Cx3cr1*^*Cre*^:*Usp18*^*fl/fl*^ mice at an age of 4 months (Fig. 2a, b) and 8 months (Fig. 2c, d). To analyze white matter integrity, we performed high-resolution (11.7 T) diffusion tensor imaging (DTI). We calculated the fractional anisotropy (FA) values, permitting an exploration of the orientation coherence of axons in this fiber bundle. We found that the FA values were reduced in the corpus callosum, the internal and external capsule of *Cx3cr1*^*Cre*^:*Usp18*^*fl/fl*^ mice (cf. *Usp18*^*fl/fl*^ controls), suggesting diminished structural integrity of the white matter in 4- and 8-month old animals (Fig. 2e). Additionally, we found increased numbers of cells that had incorporated myelin and thereby indicate damage to the myelin sheaths (Fig. 2f). Together, these findings point to a reduction in myelination or even to a loss of fibers in *Cx3cr1*^*Cre*^:*Usp18*^*fl/fl*^ mice [2, 17].

**Fig. 3:**
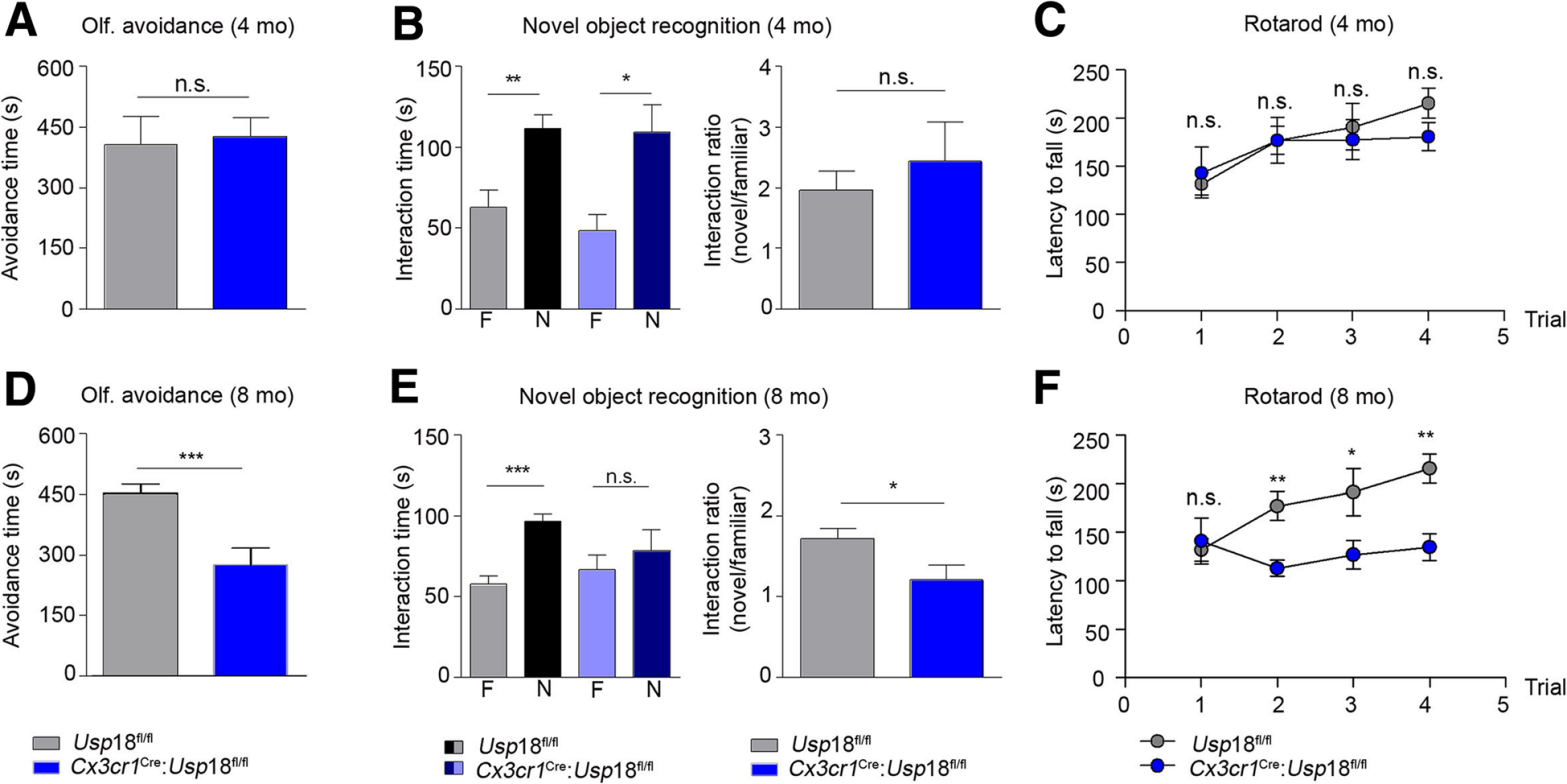
Gradual behavioral impairment in *Cx3cr1*^*Cre*^:*Usp18*^*fl/fl*^ mice. **a**, **d** Olfactory avoidance test. The time animals spent away from the odorant zone was recorded. **b**, **e** Novel object recognition. The time a mouse spent investigating a familiar (**f**) or novel (N) object was recorded. The object interaction ratio was defined as the difference in exploration time for the novel object divided by the exploration time for the familiar object. **c**, **f**) Rotarod. Graphed is the latency to fall off the rod during accelerating speed (4–40 r.p.m). For all three tests, performance of *Usp18*^*fl/fl*^ and *Cx3cr1*^*Cre*^:*Usp18*^*fl/fl*^ animals was compared when they had reached 4 and 8 months of age. Asterisks indicate significant differences (*P < 0.05, **P < 0.01 and ***P < 0.001, n.s. = not significant)

**Fig. 4:**
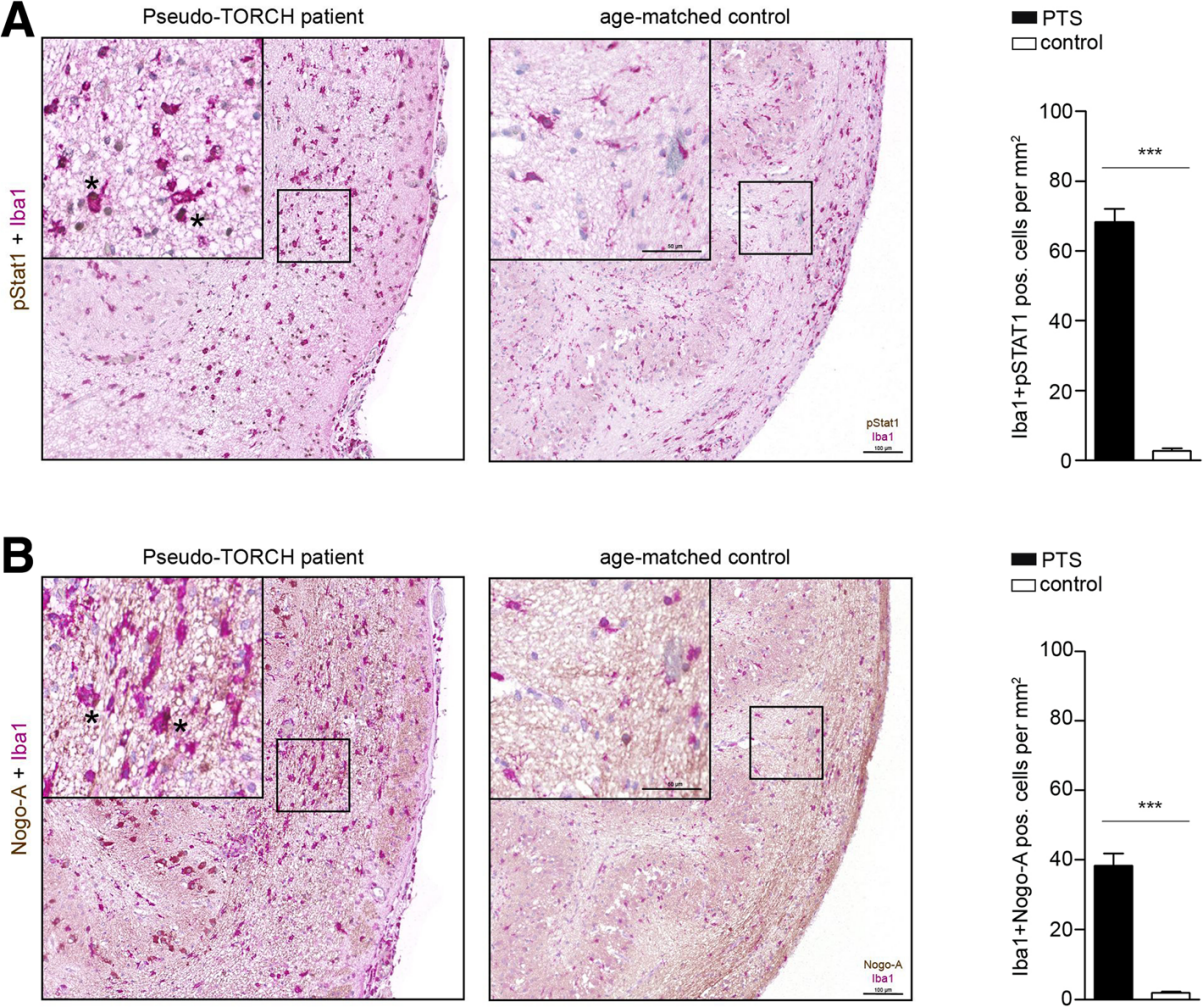
Microgliosis in white matter of Pseudo-TORCH patients. **a** Histology of white matter in Pseudo-TORCH patients (*n* = 3) and age-matched controls (*n* = 3) (**b**). Primary antibodies were used against Iba1, pStat1 and Nogo-A. Quantification of cells is shown next to the respective histological images. Significant differences were determined by an unpaired *t*-test or Mann-Whitney *U*-test and marked with asterisks (***P < 0.001 versus controls). Bars represent means ± s.e.m. Scale bars = 50 μm, 100 μm

Deterioration of white matter tracts, affecting brain structural (SC) and functional connectivity (FC) is often paralleled by behavioral declines [3, 6, 8]. We therefore tested *Cx3cr1*^*Cre*^:*Usp18*^*fl/fl*^ mice and *Usp18*^*fl/fl*^ littermate controls in different behavioral paradigms. While mice lacking *Usp18* in microglia performed normal in the odor avoidance test at 4 months of age (Fig. 3a), 8-month old *Cx3cr1*^*Cre*^:*Usp18*^*fl/fl*^ mice showed severely impaired olfaction (Fig. 3d). Similarly, learning and recognition memory was fully intact at 4 months of age (Fig. 3b) but decreased when *Cx3cr1*^*Cre*^:*Usp18*^*fl/fl*^ mice were 8-month old compared to age-matched *Usp18*^*fl/fl*^ control mice (Fig. 3e). Rotarod performance, which measures motor coordination and motor learning, was also significantly impaired in 8-month old *Cx3cr1*^*Cre*^:*Usp18*^*fl/fl*^ mice (Fig. 3f) with no deficits in 4 months old mice (Fig. 3c). In addition to the indicated mouse model we investigated brainstem tissue samples from three PTS patients with loss-of-function recessive mutations of *USP18* [12]. Immunohistochemistry showed increased STAT1 phosphorylation in microglia of PTS patients when compared to age-matched control tissue (Fig. 4a). In patients’ material there were also more microglial cells, which engulfed cells positive for Nogo-A (Fig. 4b), which represents an oligodendroglial marker [11].

The data presented here indicate that in myeloid-specific *Usp18* knockout animals, microglia in the white matter were not only activated, but also caused advancing damage to this structure with subsequent behavioral impairment of the animals. *USP18*-deficiency in humans belongs to a group of genetic disorders that are collectively termed type I interferonopathies. These disorders are first characterized by the persistent up-regulation of type I interferon signaling [16]. There have been at least seven possible cellular mechanisms described, which result in sustained activation of interferon signaling [16]. One of them, PTS, is a group of not so well-defined genetic diseases, which can originate from USP18 deficiency. We found that microglia in PTS patients displayed not only enhanced type I IFN signaling, but also close contact to oligodendroglia. A direct interaction might indicate that activated microglia, as suggested by their focally elevated cell density together with altered morphological properties inflict damage to oligodendroglia. This strongly resembles the white matter damage observed in *Cx3cr1*^*Cre*^:*Usp18*^*fl/fl*^ mice. Type I interferon can be regarded as a neurotoxin if its levels are not tightly controlled. Accordingly, experiments undertaken in mice demonstrate that overexpression of interferon in the CNS results in neuropathology reminiscent of that seen in certain type I interferonopathies [1, 10]. In the case of PTS, but also in the case of type I IFN overexpression, damage to the white matter seems to be prevalent [5, 12]. It is still unclear what the type I IFN source is in the context of interferonopathies. Likewise it is enigmatic which signals are responsible for microglia activation in the white matter. The escalating spiral of white matter damage might be initiated by type I IFN that is induced in microglia via stimulator of interferon genes (STING), and this IFN likely influences the microglial phenotype in an autocrine and paracrine fashion [13].

The white matter specificity of the USP18 effect on microglia is of particular interest and further developments in this area may have implications for an entire range of neurological disorders in which there is a preponderance of white matter pathology.

## Data Availability

The datasets used and/or analysed during the current study are available from the corresponding author on reasonable request.

## Abbreviations

DTI: Diffusion tensor imaging
IFN: Interferon
ISG: Interferon-stimulated gene
MRI: Magnetic resonance imaging
NOR: Novel Object Recognition
PTS: Pseudo-TORCH syndrome
RT: Room temperature
STAT1: Signal transducer and activator of transcription 1
STING: Stimulator of interferon genes
USP: Ubiquitin-specific protease
